# Novel Pathogenic Variants in *POLR3K* Cause POLR3-Related Leukodystrophy

**DOI:** 10.1155/2024/8807171

**Published:** 2024-07-31

**Authors:** Stefanie Perrier, Julia Macintosh, Agata D. Misiaszek, Gabrielle Lambert, Kether Guerrero, Luan T. Tran, Christoph W. Müller, Tomi Pastinen, Gustavo H. B. Maegawa, Isabelle Thiffault, Geneviève Bernard

**Affiliations:** ^1^ Department of Neurology and Neurosurgery McGill University, Montréal, Quebec, Canada; ^2^ Child Health and Human Development Program Research Institute of the McGill University Health Centre, Montréal, Quebec, Canada; ^3^ Structural and Computational Biology Unit European Molecular Biology Laboratory, Heidelberg, Germany; ^4^ Faculty of Biosciences Heidelberg University, Heidelberg, Germany; ^5^ Department of Pediatrics McGill University, Montréal, Quebec, Canada; ^6^ Genomic Medicine Center Children's Mercy Hospital, Kansas City, Missouri, USA; ^7^ University of Missouri Kansas City School of Medicine, Kansas City, Missouri, USA; ^8^ Departments of Pediatrics and Genetics Columbia University Irving Medical Center, New York, New York, USA; ^9^ Department of Pathology and Laboratory Medicine Children's Mercy Hospital, Kansas City, Missouri, USA; ^10^ Department of Human Genetics McGill University, Montréal, Quebec, Canada; ^11^ Department of Specialized Medicine Division of Medical Genetics Montreal Children's Hospital and McGill University Health Centre, Montréal, Quebec, Canada

**Keywords:** case report, hypomyelination, Pol III, POLR3K, POLR3-related leukodystrophy, RNA polymerase III, RPC10

## Abstract

POLR3-related hypomyelinating leukodystrophy (POLR3-HLD) is a rare inherited neurological disorder caused by biallelic pathogenic variants in specific genes encoding subunits of RNA polymerase III (Pol III). Here, we report the third patient worldwide with pathogenic variants in *POLR3K* and clinical features consistent with POLR3-HLD. The female patient presented with mild intellectual and behavioural disturbances in childhood, as well as growth delay, with brain MRI revealing diffuse hypomyelination and a pattern consistent with POLR3-HLD. In adolescence, she manifested minor motor dysfunction. Next-generation sequencing revealed a paternally inherited missense variant in *POLR3K* (c.322G>T; p.D108Y) and a maternally inherited large deletion, spanning approximately 17.8 kb from chr16:30,362-48,162. The missense variant is located at the C-terminus position of the protein and is predicted to impair residue interactions and cause steric interference in enzyme conformational changes. The large deletion encompasses the third and last exon of *POLR3K*, leading to a likely amorphic truncated protein product lacking the final 42 amino acids from the total 108 amino acid–length protein. Studies of RNA-level expression showed a significant reduction in the levels of *POLR3K* RNA in the patient compared to the control. In considering whether the transcriptional function of Pol III was affected, the expression of several Pol III-transcribed RNAs was measured, where the levels of several distinct tRNAs were significantly reduced in the patient while the expression of other RNA transcripts was not decreased, suggesting that Pol III retains partial function. This study provides further evidence for the association of pathogenic variants in *POLR3K* with POLR3-HLD, expanding the spectrum of pathogenic variants in genes encoding for Pol III subunits associated with this disease.

## 1. Introduction

RNA polymerase III-related hypomyelinating leukodystrophy (POLR3-HLD) is a rare genetic disorder caused by biallelic pathogenic variants in specific genes encoding subunits of the transcription enzyme RNA polymerase III (Pol III), and one of the most common hypomyelinating leukodystrophies (HLDs) [1–9]. POLR3-HLD is typically associated with a trio of clinical findings, including hypomyelination, hypodontia, and hypogonadotropic hypogonadism, and it is thereby often referred to as 4H leukodystrophy. Patients with POLR3-HLD typically present in early childhood and experience progressive neurological features that involve motor abnormalities including cerebellar dysfunction with variable pyramidal and extrapyramidal signs, intellectual disability, and cognitive impairment, as well as non-neurological features including myopia, short stature, abnormal puberty, and/or dental abnormalities [10–12]. The MRI pattern seen in patients with POLR3-HLD is specific, involving diffuse hypomyelination (i.e., hyperintensity of the white matter on T2-weighted images and hyper/hypo/isointensity of the white matter on T1-weighted images, compared to grey matter structures) with preservation of certain structures, with or without cerebellar atrophy and thinning of the corpus callosum [13–15].

Pol III is a 17-subunit complex responsible for transcribing several different noncoding RNAs, and of its subunit-encoding genes, variants in *POLR3A* and *POLR3B* were first discovered to cause POLR3-HLD, followed by *POLR1C* and, more recently, *POLR3K* and *POLR3D* [1–7]. The first study implicating *POLR3K* as a causal gene in 2018 described two unrelated males with hypomyelination and clinically progressive neurological features [6]. These patients harboured the same homozygous variant in *POLR3K*, associated with decreased expression of certain Pol III transcripts [6]. This report describes the third patient worldwide with biallelic pathogenic variants in *POLR3K* and a clinical and radiological phenotype consistent with POLR3-HLD.

## 2. Materials and Methods

### 2.1. Informed Consent

This research was approved by the Montreal Children's Hospital and McGill University Health Centre Research Ethics Boards (11-105-PED; 2019-4972) and the Children's Mercy Institutional Review Board (study #11120514) and has been performed following the ethical standards defined in the Declaration of Helsinki. Informed consent was obtained from the participant/parents.

### 2.2. Clinical and Genetic Investigations

The available medical records and MRIs were reviewed. Clinical exome sequencing data were obtained and analyzed based on the *American College of Medical Genetics* (ACMG) *Standards and Guidelines for the Interpretation of Sequence Variants* [16], leading to the discovery of a *POLR3K* missense variant, which was confirmed by Sanger sequencing to be paternally inherited. To resolve the large deletion, genome sequencing was completed as previously described [17] using DNA extracted from saliva collected using the Oragene OG-500 kit, according to the manufacturer's instructions. The software Bionano VIA™ v.7.0 (previously NxClinical) was used for copy number variant (CNV) calling from genome sequencing BAM files, which detects changes by calculating read depth alterations, determined by comparison with similar sequence coverage profiles in a sequencing cohort. Integrative Genomics Viewer (IGV) software was used to visualize CNVs, which were reviewed for pathogenicity via presence in the Database of Genomic Variants (DGV), ClinVar, or the gnomAD structural variant callset, with exclusion if indicated to overlap with regions frequently affected by CNVs (MAF ≥ 1%). Further, CNVs were evaluated for pathogenicity based on the technical standards for interpretation of constitutional CNVs from the ACMG guidelines [16] and the Clinical Genome Resource (ClinGen).

### 2.3. Protein Modelling

Structural analysis of the human Pol III enzyme was achieved using the UCSF ChimeraX molecular visualization program [18]. The presented structures are PDB:7D58 for the “inside funnel” conformation [19] and PDB:7AEA for the “outside funnel” conformation [20]. In the close-up panels, residues shown in stick representation are within 5 Å from the D108 residue. Simulation of the D108Y mutation was achieved using the swapaa command in UCSF ChimeraX.

### 2.4. RNA Expression in Fibroblasts

#### 2.4.1. Cell Culture

Fibroblasts from the described individual and a healthy control were cultured in DMEM containing 10% fetal bovine serum (FBS, Wisent Bio Products, Quebec) and maintained at 37°C with humidification and 5% CO_2_.

#### 2.4.2. RNA Extraction and RT-qPCR

Fibroblasts were lysed in TRIzol reagent (Thermo Fisher), and total RNA was extracted using a miRNeasy kit (Qiagen), following the manufacturer's instructions and with on-column DNase I treatment (Qiagen). RNA concentration and quality were assessed on an ND-1000 Nanodrop Spectrophotometer. For each sample, 500 ng of RNA was reverse transcribed with M-MLV reverse transcriptase (Promega) and a 1:1 mix of Oligo(d)T (Thermo Fisher) and random hexamer primers (IDT). Primers were designed to span exon–exon junctions using the Primer-Blast NCBI and OligoAnalyzer IDT software (Table S1). The primers used to measure *POLR3K* mRNA expression were designed in Exon 1 of the protein-coding transcript (ENST00000293860.6). All primers were validated using a 1:1 mix of control cDNA and cDNA from the affected individual to meet MIQE guidelines [21, 22]. cDNA was amplified using a Roche LightCycler® 96 instrument using the following parameters: preincubation (95°C for 180 s), 3-step amplification (95°C for 10 s, 60°C for 30 s, and 72°C for 30 s; 45 cycles), and melting (95°C for 10 s, 65°C for 60 s, and 97°C for 1 s), and expression was assessed using SsoAdvanced Universal SYBR Green Supermix (Bio-Rad Laboratories). Gene expression of each biological replicate (*n* = 3) was analyzed in technical triplicates using the ΔΔCt method with a 1:40 cDNA dilution and normalized to three reference genes (*PGK1*, *GUSB*, and *TFRC*). Expression is presented as relative to the control cell line.

#### 2.4.3. Statistical Analysis

Data analysis was performed using GraphPad Prism 9.1.1 using quantifications from three biological replicates. Statistical analysis was performed using a Student's *t*-test to compare between two groups (Table S2). Statistical significance was set at ^∗^*p* < 0.05. Data are shown as mean ± SEM.

## 3. Results

### 3.1. Clinical Presentation

#### 3.1.1. Perinatal History

Following an uneventful pregnancy, the female patient was born at 36 weeks via caesarean section in the context of failure to progress. No perinatal distress or neonatal intensive care unit admissions were reported, and she was discharged on the third day of life. The patient was the only child of nonconsanguineous, healthy parents with no family history of intellectual disabilities, neurological conditions, or congenital malformations on both the maternal and paternal sides. The mother is of Brazilian descent, and the father is of Brazilian and Japanese descent.

#### 3.1.2. Early Psychomotor Development

Early developmental milestones were achieved at appropriate ages. Regarding early motor development, she sat at age 6 months and walked independently at 12 months. Her early fine motor skills developed adequately. The patient said her first words between 12–15 months, and spoke two-to-three-word sentences by 18–24 months. No speech concerns were noted; the patient is multilingual and fluent in three languages. No concerns were noted regarding social interactions with peers in early childhood. She was fully toilet-trained by 2.5–3 years of age. As a young child, she participated in appropriate activities for her age, including drawing and biking.

#### 3.1.3. Motor Manifestations

In early adolescence, at 12 years of age, fine motor skill difficulties were first noted, including new progressive clumsiness such as dropping objects and difficulty using utensils properly. Four years later, at the age of 16 years, the patient started to show signs of gross motor difficulties, presenting as poor coordination while running, leading to occasional falls. In terms of functional status, the patient remained at Level I on the Gross Motor Function Classification System (GMFCS), Manual Ability Classification System (MACS), and Communication Function Classification System (CFCS) scales throughout her childhood and teenage years.

#### 3.1.4. Neurological Examinations

From ages 12 to 17 years, neurological examinations showed evidence of ataxia with difficulty performing tandem gait as well as saccadic visual pursuit in all directions of gaze. Coordination was reported as normal throughout childhood and adolescence, with no evidence of dysmetria and dysdiadochokinesia. No dysarthria, pyramidal, or extrapyramidal signs were reported. Reported head circumference measurements between the ages of 10 and 17 years remained around the 15th percentile.

#### 3.1.5. Nerve Conduction Studies

Nerve conduction studies performed at 10 years of age showed normal results with no evidence of neuropathy.

#### 3.1.6. Neuropsychological Assessment

At the age of 8 years old, the patient started to present signs of attention difficulties, anxiety, and behavioural changes in the form of stubbornness and irritability. She was later diagnosed with attention deficit hyperactivity disorder (ADHD), inattentive subtype, at the age of 12 years. She initially responded well to psychostimulant medication. At 13 years old, her sustained attention and executive skills on the Conners' Continuous Performance Test (CPT)-II test [23] were reported to be within the average range. At her last visit at 17 years old, the patient was still medicated for ADHD.

From childhood to adolescence, the patient experienced learning difficulties predominantly affecting her language. She demonstrated a moderate language disorder, both receptive and expressive, leading to difficulties with reading and writing skills. Between assessments at 8 and 13 years old, her intelligence quotient (IQ) remained overall stable in the low-average range as per the Wechsler Intelligence Scale for Children 4th and 5th editions (WISC-IV [24] and WISC-V [25]). More specifically, her Processing Speed Index remained in the average range. Her Word Memory Index went from the borderline-to-low-average range at 8 years old to the average range at 13 years old, representing an area of relative strength. At 13 years of age, her Visual Capacity Index and Verbal Comprehension Index were in the average range. However, the Fluid Reasoning Index fell in the borderline range and was noted to be an area of greater difficulty. At the age of 15, her academic evaluation on the Woodcock–Johnson-IV (WJ-IV) test [26] fell in the low-average-to-average range in reading, writing, and mathematics, with reading comprehension and oral reading scores below grade-level expectations. This represented a slight decrease from her performance at the age of 11 years old, where her WJ-IV fell in the average range with similar prominent difficulty pertaining to reading comprehension. Additionally, at age 15 years, the Kaufman Assessment Battery for Children (KABC-II) [27] showed a nonverbal score within the average range for age; however, the patient was noted to have significant difficulty in the story completion subtest when telling meaningful stories using pictures, appearing in line with her documented language deficits. Results from the current cognitive assessment revealed adequate nonverbal reasoning skills. The Test of Auditory Processing Skills (TAPS, 3rd ed.) [28] revealed deficits in auditory comprehension and reasoning (Cohesion Index). The patient's auditory memory fell slightly below the average range, and her greatest difficulty was shown in recalling longer sentences accurately.

#### 3.1.7. Non-neurological Features

Regarding non-neurological features, the patient also experienced growth abnormalities. She had growth delay during early to middle childhood and was treated with growth hormone (GH) for a brief 8-month period at 8 years of age, but stopped due to behavioural changes and after leukodystrophy findings were first discovered on a standard brain MRI during GH therapy assessment. Though her short stature persisted, she maintained growth velocity, and at 12 years of age, her IGF-1 levels were in the normal range, with increased IGFBP-3 levels. From ages 11 to 17 years, her height and weight *z*-score values fell below the general population, with an average height *z*-score of −1.581 (SD ± 0.205) and an average weight *z*-score of −1.420 (SD ± 0.297) based on yearly measurements.

She experienced normal puberty, with thelarche and pubarche at age 12 and menarche at age 13. She also had normal levels of TSH, cortisol, FSH, LH, ACTH, estradiol, and prolactin.

She had ophthalmic abnormalities, including bilateral posterior polar cataracts (treated surgically at age 14 years) and photosensitivity. Visual acuity remained normal.

Additionally, she had gastrointestinal (GI) problems, including frequent abdominal pain with vomiting throughout childhood. Upper GI endoscopy was diagnostic for gastritis and cobble-stoning of the gastric mucosa, with a biopsy significant for lymphoid hyperplasia in the duodenal mucosa. By 11 years of age, the patient developed lactose intolerance, which was clinically diagnosed, as improvements in symptoms with the use of probiotics and the avoidance of dairy products were noticeable. In adolescence, intermittent fecal incontinence was reported, which improved over time. Constipation was also noted.

Her direct bilirubin levels were persistently high, and a homozygous UGT1A1∗28 polymorphism in the *UGT1A1* gene (c.-53_-52insTA, rs3064744) was found through clinical investigations. This gene encodes the enzyme bilirubin uridine diphosphate glucuronosyltransferase, and the UGT1A1∗28 variant denotes the presence of an additional TA repeat in the gene's promoter TATA box sequence, known to be associated with hyperbilirubinemia and Gilbert's syndrome [29, 30].

### 3.2. MRI Features

**Figure 1:**
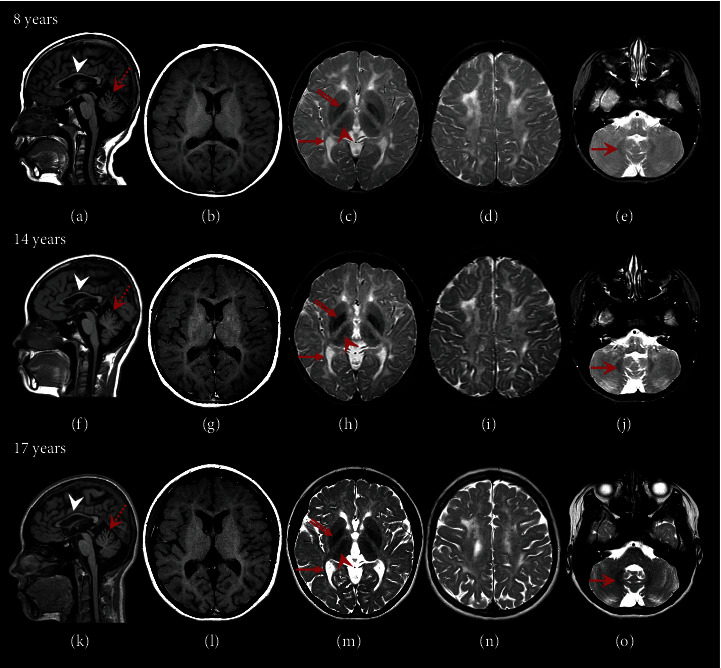
MRI features. Serial neuroimaging at Ages 8, 14, and 17 years, including sagittal T1- (Column 1: (a), (f), and (k)), axial T1- (Column 2: (b), (g), and (l)), and axial T2- (Column 3: (c), (h), and (m); Column 4: (d), (i), and (n); Column 5: (e), (j), and (o)) weighted MRI images. Diffuse hypomyelination is evident: there is hyperintensity of the white matter on T2-weighted imaging (c, d, h, i, m, n), together with a variable signal of the white matter on T1-weighted imaging, with hyperintense/isointense/slightly hypointense areas of the white matter (b, g, l), compared to grey matter structures. Relative myelin preservation (T2-hypointensity) is seen in the optic radiations (red arrows in (c), (h), and (m)), the anterolateral nucleus of the thalamus (red arrowhead), the globus pallidus (red double-lined arrow), and the dentate nucleus (red arrow in (e), (j), and (o)), with overall mildly improved myelination over time. Mild cerebral atrophy is evident by Age 17. A thin corpus callosum (white arrowheads) and mild superior cerebellar vermis atrophy (red dashed arrow) are demonstrated, without significant levels of progression over time (a, f, k).

Brain MRI at 8 years of age revealed hypomyelination, with T2-weighted hyperintensity of the white matter and variable white matter signal on T1-weighted images, with areas of hyperintensity, isointensity, and mild hypointensity, compared to grey matter structures (Figure 1). In a pattern consistent with POLR3-HLD, there was relative preservation of myelination of the optic radiations, anterolateral nucleus of the thalamus, globus pallidus, and dentate nucleus. She also had thinning of the corpus callosum and mild superior cerebellar vermis atrophy (Figures 1(a), 1(f), and 1(k)). Besides mild improvement of myelination over time (Figures 1(c), 1(e), 1(h), 1(j), 1(m), and 1(o)) and mild cerebral atrophy on her most recent MRI at 17 years of age (Figure 1(n)), her brain MRIs appeared stable throughout childhood to late adolescence.

### 3.3. Molecular Genetics

#### 3.3.1. Variant Identification

Genetic investigations began with secondary analysis of clinical exome sequencing data on a research basis according to ACMG guidelines [9], leading to the identification of a missense variant and a large deletion in trans (PM3). A paternally inherited missense variant in *POLR3K*, c.322G>T; p.D108Y (NM_016310.5), was first detected. The p.D108Y variant had not been reported in population databases including gnomAD (v2.1.1; https://gnomad.broadinstitute.org/) (PM2), and in support of the candidate variant pathogenicity, two variants at the same position (p.D108E and p.D108H) only had a single heterozygous individual reported in the control population, demonstrating that the residue is associated with very low allele frequency for variants and without homozygous individuals reported in the control population. In addition, the residue is highly conserved between species. This variant is predicted to be pathogenic by several in silico prediction softwares, and the amino acid residue is highly conserved between species (PP3). Following Sanger sequencing cosegregation analysis of the missense variant, a large maternally inherited deletion was also suspected (Figure S1). Genome sequencing resolved the deletion to be approximately 17.8 kb, located from chr16:30,362-48,162 (GRCh38). Notably, due to the repetitive regions near the downstream breakpoint, these deletion coordinates serve as an estimate owing to the limitations of the genomic sequencing coverage and may extend into the region of 16p13.3. The upstream deletion breakpoint is located in the final 3′ intron of *POLR3K*, leading to the deletion of the third and final exon of the gene, with a predicted loss of the last 42 amino acids from the C-terminal of the full 108 amino acid length wild-type protein (~60% truncated protein), thereby predicted to cause loss of function (PVS1) (NM_016310.5: c.200_327del; p. E67Gfs∗18). The deleted region following the *POLR3K* gene is largely intergenic and nontranslated, only containing a portion of the long noncoding RNA gene ENSG00000260803 prior to the downstream breakpoint. No similar deletion is reported in the gnomAD and DGV databases. Figure S1 illustrates the location of both variants.

#### 3.3.2. Protein Modelling

**Figure 2:**
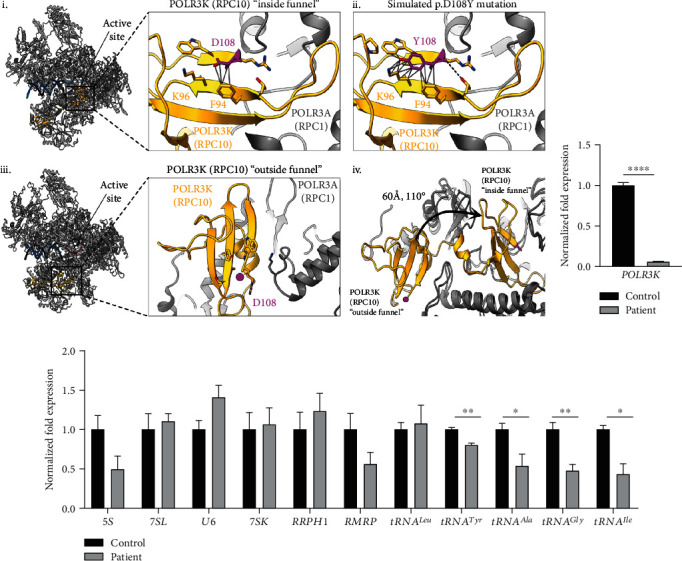
Functional implications of the *POLR3K* variants. (a) Structural mapping of the *POLR3K* (RPC10) c.322G>T; p.D108Y variant onto the human Pol III enzyme complex. (i) Structure of the human Pol III complex (PDB:7D58) with the POLR3K (RPC10) subunit (yellow) shown in the “inside funnel” conformation. (ii) Simulation of the D108Y variant (magenta) demonstrating possible interactions with neighboring residues (F94 and K96), with hydrogen bonds denoted by black dotted lines and contacts/clashes denoted by solid grey lines. (iii) Human Pol III complex structure (PDB:7AEA) with the POLR3K (RPC10) subunit (yellow) shown in the “outside funnel” conformation. The putative location of the D108 residue (not modelled in the structure) is marked with a magenta dot. (iv) Illustration of conformational change of the POLR3K (RPC10) subunit when inserting into the funnel and active site of Pol III, denoted with a black arrow. (b, c) Normalized fold expression of *POLR3K* and Pol III transcripts in the patient relative to a healthy control, as assessed by RT-qPCR. Statistical analysis was performed using a Student's *t*-test to compare between two groups with statistical significance set at ^∗^*p* < 0.05. (b) Normalized fold expression of *POLR3K* demonstrates very low expression of the subunit in the patient compared to a control. (c) Relative gene expression of Pol III-transcribed genes, demonstrating a significant decrease in expression of *tRNA^Tyr^*, *tRNA^Ala^*, *tRNA^Gly^*, and *tRNA^Ile^*. The expression of other transcripts (*5S*, *7SL*, *U6*, *7SK*, *RRPH1*, *RMRP*, and *tRNA^Leu^*) was not significantly decreased, suggesting some residual Pol III function. ^∗^*p* < 0.05; ^∗∗^*p* < 0.01; ^∗∗∗∗^*p* < 0.0001.

As the *POLR3K* missense variant c.322G>T (p.D108Y) is located at the final C-terminal position of *POLR3K* directly adjacent to the termination codon, we sought to investigate its impact on the protein level by modelling the variant onto the human Pol III enzyme [20]. POLR3K (also known as RPC10) is a mobile subunit of the Pol III complex with the ability to adopt two different conformations, either located inside or outside of the polymerase funnel, dependant on the transcriptional state (Figure 2(a) i, iii). For the conformation in which POLR3K is inserted inside the polymerase funnel (thought to be associated with an active transcription elongation state), the C-terminal domain of the POLR3K subunit is located near the active site of the Pol III enzyme. Simulation of the D108Y variant onto the subunit in this conformation (Figure 2(a) ii) demonstrates that steric clashing may occur between neighboring residues such as K96 or that stacking interactions could occur with the F94 residue. When in the outside conformation, the D108Y variant is located on the surface of the complex and does not form contacts with other residues (Figure 2(a) iii); however, as the subunit must undergo a large conformational change in order to insert into the funnel (Figure 2(a) iv), it remains possible that the mutation could sterically interfere with this process, causing disruptions to RNA cleavage and/or termination.

#### 3.3.3. Functional Studies

To determine the consequences of the variants on *POLR3K* expression and Pol III transcriptional function, we compared the RNA expression levels of *POLR3K* and several Pol III-transcribed genes in fibroblasts from the patient and a healthy control. The level of *POLR3K* RNA expression was significantly decreased in the patient (93.6% reduced, *p* = 0.000011) compared to the healthy control (Figure 2(b) and Table S2), thus confirming that both mutations impact the RNA expression of the subunit. We also tested the expression of several Pol III transcripts, where the levels of several distinct tRNAs, including *tRNA^Tyr^*, *tRNA^Ala^*, *tRNA^Gly^*, and *tRNA^Ile^*, were significantly reduced (Figure 2(c) and Table S2). The other measured RNA transcripts (*5S*, *7SL*, *U6*, *7SK*, *RRPH1*, *RMRP*, and *tRNA^Leu^*) were not significantly decreased, indicating that Pol III retains partial transcriptional function.

## 4. Discussion

In sum, this study further expands the spectrum of variants in Pol III genes associated with hypomyelination, demonstrating the importance of proper Pol III complex function during myelin development. The patient we describe with biallelic pathogenic variants in *POLR3K*, including a missense variant and a large deletion, exhibits clinical and radiological features consistent with POLR3-HLD. Of the Pol III subunits implicated in POLR3-HLD disease, pathogenic variants in *POLR3K* are rare, with only two patients previously described in the literature, both males from two unrelated consanguineous families who both harboured the same homozygous variant (c.121C>T; p.R41W) [6]. These patients demonstrated an early disease onset within the first year of life, and typical neurological features associated with POLR3-HLD, including cerebellar signs, dystonia, and pyramidal features. The degree of neurological involvement at an early age was more severe compared to the patient we report, as they first experienced the onset of nystagmus within the first 2 years of life, had motor decline leading to the loss of head hold control in childhood, and had severe spasticity before age 10 years. They also had difficulties with language acquisition, with one patient being nonverbal and the other only speaking isolated words by age 2 years, with loss of language at age 6 years. Our patient presents with a milder phenotype, exhibiting learning difficulties throughout childhood, evolving to cognitive and mild motor impairments in late adolescence. She did not display severe spasticity, other pyramidal features, or dystonia. She also did not experience difficulties with language or motor acquisition.

In a large cohort study of patients with POLR3-HLD and variants in *POLR3A* or *POLR3B* [10], the majority of patients presented with motor delay or regression in early childhood; however, approximately 10% of patients had a later onset of disease with learning difficulties and development of motor impairment. The patient we describe here aligns with this milder presentation and later onset of disease. It is important to note that phenotypic differences in severity are evident within the spectrum of disease seen in POLR3-HLD, for which both mild and severe presentations have been reported [10, 11, 31–53]. Neurological features associated with POLR3-HLD typically include prominent cerebellar involvement, including gait ataxia, dysarthria, and dysmetria. Patients may also show pyramidal signs (e.g., spasticity) as well as extrapyramidal signs (e.g., dystonia) [54, 55]. As the disease progresses, loss of ambulation often leads to wheelchair dependency in adolescence, and death usually occurs in early adulthood [10]. Further, depending on the causal gene, the disease course of patients with POLR3-HLD broadly follows a pattern of severity, with the most severe course associated with *POLR1C* variants, followed by those in *POLR3A* and *POLR3B* [10, 11]. Only one patient has been reported with variants in *POLR3D* who demonstrated a typical presentation of later-onset POLR3-HLD [7]. As discussed above, there is phenotypic variability in those with variants in *POLR3K*, including the earlier onset of disease described by Dorboz et al. [6] in two patients and the report of this patient with a later onset of disease. Further, most patients (> 90%) harbour variants in *POLR3A* and *POLR3B*, and fewer patients (< 10%) are reported to harbour variants in *POLR1C*, *POLR3D*, and *POLR3K* [1–7, 9–11].

The patient we describe also experienced non-neurological features seen in POLR3-HLD, including growth delay and ophthalmic abnormalities. Further, she experienced GI symptoms, a feature not usually associated with the typical POLR3-HLD phenotype caused by variants in other Pol III subunits. Interestingly, other patients with *POLR3K* variants also experienced digestive dysfunction, and in a *Polr3b* zebrafish model involving a genetic deletion thought to impact Polr3b-Polr3k interactions, digestive development was significantly impaired [6, 56]. This may suggest that tissue-specific developmental defects are caused by distinct impairments of Pol III function involving the POLR3K subunit. However, our patient's clinical investigations demonstrated she harboured a variant in the *UGT1A1* gene associated with increased bilirubin. The homozygous UGT1A1∗28 allele has a relatively high prevalence in the general population, varying from 1% to 23% depending on ethnicity, with many individuals remaining asymptomatic [57].

The patient's brain MRI pattern was consistent with that typically seen in POLR3-HLD, involving diffuse hypomyelination and relative preservation of myelin in specific structures, with or without thinning of the corpus callosum and mild cerebellar atrophy [10, 13, 15, 58]. Over time, the MRI pattern appeared stable, without evidence of significant progressive changes from childhood to late adolescence. In contrast, the two other published patients with variants in *POLR3K* demonstrated progressive atrophy of supratentorial and infratentorial structures, with white matter atrophy, between early and late childhood [6]. However, given the spectrum of disease severity often seen in patients with POLR3-HLD, this is not unexpected, as rates of disease progression vary between patients with variants in the same gene [10, 11]. Moreover, it is known that typical POLR3-HLD disease presentations are not strictly driven based on the mutated gene but may be associated with different progression rates of both clinical and MRI features. This could be further explained by the fact that Pol III is an enzyme complex, where different pathogenic variants may impair subunit function through alternative processes and/or severity, with various levels of complex hypofunction associated with progressive disease phenotypes.

The patient we described harboured a large deletion and a novel missense mutation in *POLR3K*. As the large deletion results in the loss of over a third of the total gene product, the resulting transcript is likely amorphic and subject to degradation. Large deletions have been reported in other instances of POLR3-HLD, including those spanning exonic regions of *POLR3B* [59]. Protein modelling of the missense variant shows it is located at the end of the C-terminus, a region within the active site of the enzyme. As such, it may disrupt the conformational changes that the POLR3K domain undergoes when changing between active transcription states. Additionally, studies of *POLR3K* RNA expression in fibroblasts demonstrated very low levels in the patient compared to a control, likely due to degradation of the RNA transcripts via aberrant RNA decay mechanisms. Moreover, as complete loss of Pol III function is incompatible with life, we hypothesize that the protein product containing the missense variant retains partial subunit function. Studies in *Saccharomyces cerevisiae* have noted that the POLR3K analogous subunit C11 is essential, with specific roles in the facilitated recycling of the Pol III complex and RNA cleavage [60]. Though our results demonstrate very low *POLR3K* RNA expression, the patient displayed a relatively mild phenotype compared to others with POLR3-HLD, including the two other reported patients with variants in *POLR3K* [6]. It is important to note that RNA-level expression of the subunit was measured, which may not accurately represent the expression of the protein, as the relationship between RNA transcript levels and downstream posttranslation protein levels remains complex [61]. RNA levels were also measured in fibroblasts, which may not be indicative of protein expression in other tissues, including the brain. Further, when considering the transcriptional function of the Pol III complex, our results show that the expression of four out of five distinct tRNA species measured were decreased (i.e., *tRNA^Tyr^*, *tRNA^Ala^*, *tRNA^Gly^*, and *tRNA^Ile^*). At the same time, several other Pol III-transcribed RNAs did not show significantly decreased levels (i.e., *5S*, *7SL*, *U6*, *7SK*, *RRPH1*, *RMRP*, and *tRNA^Leu^*), suggesting preservation of Pol III function and transcriptional activity. Compared to the two other published patients with variants in *POLR3K*, there is variability in the expression of different noncoding RNAs; in contrast to our patient, the two other patients showed significantly reduced levels of *5S* and *7SL* and normal expression of *tRNA^Ala^* and *tRNA^Gly^* [6]. Additionally, these patients did not demonstrate the significant reduction in expression of *POLR3K* subunit levels seen in our patient. As the two previous patients harboured a different missense variant and had a more severe phenotype, it is likely that alternative mechanisms of pathogenesis are occurring on a molecular level compared to the patient we describe. Overall, we hypothesize that in this patient, the pathogenic variants in *POLR3K* cause Pol III to be hypomorphic, impacting global protein expression, further leading to defects in development and the hypomyelination seen on MRI. We therefore hypothesize that the degree of Pol III complex function, and not the expression of individual subunits, leads to phenotypes increasing in severity.

## 5. Conclusion

In conclusion, the report of this 17-year-old female provides further supporting evidence for the association of POLR3K hypofunction with impaired white matter development, broadening the range of variants associated with POLR3-HLD and demonstrating the critical role of proper transcription machinery function during the important neurodevelopmental stage of myelination.

## Data Availability

The genetic variants reported in this study have been deposited in the ClinVar database (https://www.ncbi.nlm.nih.gov/clinvar/). The raw data from participants (i.e., raw genetic data and MRI data) are not publicly available to maintain anonymity.

## Supporting Information

Supporting Information Additional supporting information can be found online in the Supporting Information section. Figure S1. (a) Genome sequencing alignment of the BAM file on IGV (Integrative Genomics Viewer), illustrating the 17.8 kb deletion from chr16:30,362-48,162. (b) Visualization of the deletion breakpoint within *POLR3K* intron 2, as well as the *POLR3K* c.322G>T; p.D108Y missense variant. (c) Sanger sequencing results showing the *POLR3K* c.322G>T; p.D108Y variant, confirmed to be paternally inherited. Due to the location of the deletion on the allele in trans, only one allele containing the missense variant was amplified by Sanger sequencing in the patient and mother. Table S1. Primer sets used for RT-qPCR. Table S2. Statistical analysis results for RNA expression levels of *POLR3K* and Pol III-transcribed genes between the patient and healthy control.
