# Supplementary material for: Novel Pathogenic Variants in POLR3K Cause POLR3-Related Leukodystrophy
Source: Hum Mutat. 2024 Jul 31;2024:8807171. doi: 10.1155/2024/8807171 (PMC11919171; doi:10.1155/2024/8807171)
Supplement: Supporting Information — Additional supporting information can be found online in the Supporting Information section. Figure S1. (a) Genome sequencing alignment of the BAM file on IGV (Integrative Genomics Viewer), illustrating the 17.8 kb deletion from chr16:30,362-48,162. (b) Visualization of the deletion breakpoint within POLR3K intron 2, as well as the POLR3K c.322G>T; p.D108Y missense variant. (c) Sanger sequencing results showing the POLR3K c.322G>T; p.D108Y variant, confirmed to be paternally inherited. Due to the location of the deletion on the allele in trans, only one allele containing the missense variant was amplified by Sanger sequencing in the patient and mother. Table S1. Primer sets used for RT-qPCR. Table S2. Statistical analysis results for RNA expression levels of POLR3K and Pol III-transcribed genes between the patient and healthy control. [file 8807171.f1.pdf]

Supplementary Information

Novel pathogenic variants in *POLR3K* cause POLR3-related leukodystrophy

Stefanie Perrier, Julia Macintosh, Agata D. Misiaszek, Gabrielle Lambert, Kether Guerrero, Luan T. Tran, Christoph W. Müller, Tomi Pastinen, Gustavo H. B. Maegawa, Isabelle Thiffault*, Geneviève Bernard*

*Corresponding Authors:

Dr. Geneviève Bernard (genevieve.bernard@mcgill.ca) and Dr. Isabelle Thiffault (ithiffault@cmh.edu).

Supplementary Table 1. Primer sets used for RT-qPCR.

Gene	F Primer	R Primer
POLR3K	CTGATCGTGGAGGAGGGACAA	CTTCCGATTTGTTACCTTGCGG
5S	GCCATACCACCCTGAACGC	TATTCCCAGGCGGTCTCCC
7SL	GGAGTTCTGGGCTGTAGTGC	TTTGACCTGCTCCGTTTCCG
U6	CGCTTCGGCAGCACATATAC	TTCACGAATTTGCGTGTCAT
7SK	CGGTCTTCGGTCAAGGGTATA	GGATGTGTCTGGAGTCTTGGA
RRPH1	GTCACTCCACTCCCATGTCCC	GGGAACTCACCTCCCCGAAG
RMRP	AGGCTACACACTGAGGACTCT	GAAGCGGGGAATGTCTACGT
tRNA-Leu-CAA-1-2	CTCAAGCTTGGCTTCCTCGT	GAACCCACGCCTCCATTG
tRNA-Tyr-GTA-8-1	AGCGGAGGACTGTAGGTTCA	GATTCGAACCAGCGACCTAA
tRNA-Ala-AGC-10-1	GGGGAATTAGCTCAAGTGGTAGA	GGGCATCGATCCCACTACCT
tRNA-Gly-TCC-4-1	GTTGGTGGTATAGTGGTGAGCA	TGCGTTGGGCGGGAATC
tRNA-Ile-TAT-3-1	GGTTAGCGCGCGGTACTTAT	TTGAACTCACAACCTCGGCA
GUSB	CACCAGGGACCATCCAATACC	GCAGTCCAGCGTAGTTGAAAAA
PGK1	GAACAAGGTTAAAGCCGAGCC	GTGGCAGATTGACTCCTACCA
TFRC	TCGTGAGGCTGGATCTCAAAA	CCTTACTATACGCCACATAACCC

Supplementary Table 2. Statistical analysis results for RNA expression levels of *POLR3K* and Pol III-transcribed genes between the patient and healthy control. A Student's t-test was used to compare between two groups and statistical significance was set at *p<0.05. Significance is denoted as *p<0.05; **p<0.01, ****p<0.0001.

Gene	P-value
POLR3K	**0.000011******
5S	0.106847
7SL	0.646505
U6	0.080380
7SK	0.825977
RRPH1	0.482385
RMRP	0.149975
tRNA Leu	0.722183
tRNA Tyr	**0.002759 ****
tRNA Ala	**0.048248 ***
tRNA Gly	**0.009390 ****
tRNA Ile	**0.013110 ***

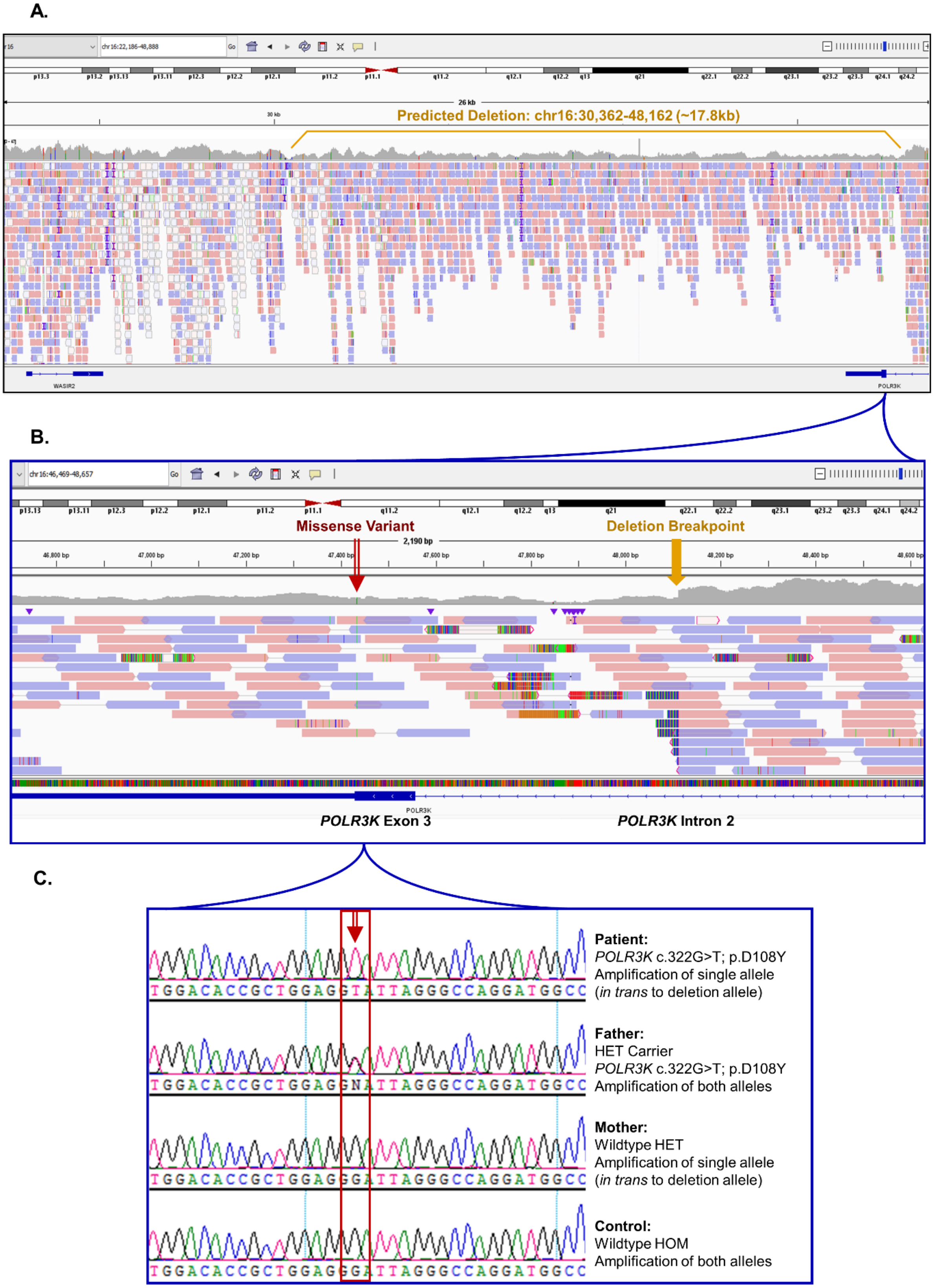

Supplementary Figure 1. (a) Genome sequencing alignment of the BAM file on IGV (Integrative Genomics Viewer), illustrating the 17.8kb deletion from chr16:30,362-48,162. (b) Visualization of the deletion breakpoint within *POLR3K* intron 2, as well as the *POLR3K* c.322G>T; p.D108Y missense variant. (c) Sanger sequencing results showing the *POLR3K* c.322G>T; p.D108Y variant, confirmed to be paternally inherited. Due to the location of the deletion on the allele *in trans*, only one allele containing the missense variant was amplified by Sanger sequencing in the patient and mother.
